# Massively Parallel DNA Sequencing Successfully Identifies New Causative Mutations in Deafness Genes in Patients with Cochlear Implantation and EAS

**DOI:** 10.1371/journal.pone.0075793

**Published:** 2013-10-09

**Authors:** Maiko Miyagawa, Shin-ya Nishio, Takuo Ikeda, Kunihiro Fukushima, Shin-ichi Usami

**Affiliations:** 1 Department of Otorhinolaryngology, Shinshu University School of Medicine, Matsumoto, Japan; 2 Department of Otolaryngology, Tsudumigaura Handicapped Children’s Hospital, Shunan, Japan; 3 Department of Otorhinolaryngology, Okayama University School of Medicine, Okayama, Japan; Tokyo Medical and Dental University, Japan

## Abstract

Genetic factors, the most common etiology in severe to profound hearing loss, are one of the key determinants of Cochlear Implantation (CI) and Electric Acoustic Stimulation (EAS) outcomes. Satisfactory auditory performance after receiving a CI/EAS in patients with certain deafness gene mutations indicates that genetic testing would be helpful in predicting CI/EAS outcomes and deciding treatment choices. However, because of the extreme genetic heterogeneity of deafness, clinical application of genetic information still entails difficulties. Target exon sequencing using massively parallel DNA sequencing is a new powerful strategy to discover rare causative genes in Mendelian disorders such as deafness. We used massive sequencing of the exons of 58 target candidate genes to analyze 8 (4 early-onset, 4 late-onset) Japanese CI/EAS patients, who did not have mutations in commonly found genes including *GJB2, SLC26A4*, or mitochondrial 1555A>G or 3243A>G mutations. We successfully identified four rare causative mutations in the *MYO15A, TECTA, TMPRSS3,* and *ACTG1* genes in four patients who showed relatively good auditory performance with CI including EAS, suggesting that genetic testing may be able to predict the performance after implantation.

## Introduction

Cochlear Implantation (CI) has been established as a standardized therapy for severe to profound hearing loss [1]. Electric Acoustic Stimulation (EAS) is a hearing implant system combining a cochlear implant and acoustic amplification technology in one device, and has recently become a standard intervention for the patients with partial deafness, defined as a mild to moderate low-frequency sensorineural hearing loss sloping to a profound hearing loss in the higher frequencies [1]. One difficult point is that outcomes of CI/EAS are variable and many factors are thought to be involved in post-implantation performance. Satisfactory auditory performance in the patients with various deafness gene mutations indicates that genetic background would be helpful in predicting performance after CI [2]. When genetic background is involved in intra-cochlear etiology, there is potential for good performance. Therefore, it is important to identify the involved region inside/outside of the cochlea by identifying the responsible gene. Decisions as to whether to undergo EAS surgery and the timing of the surgery, as well as prediction of outcome after EAS is sometimes difficult because of individual differences in progression, which is sometimes of a rather rapid nature but sometimes rather stable. One advantage of genetic testing is that the possible prognosis for hearing, i.e., progressive or not, can be predicted for individual patients.

Etiological studies have shown genetic disorders to be a common cause of deafness, but difficulty lies in the fact that deafness is an extremely heterogenous disorder.

Invader-based multi-gene screening for 13 genes/46 mutations commonly found in Japanese, identified the responsible mutations in approximately 30% of deafness patients [3], accelerating the clinical application of gene screening. However, the etiology of the rest of the patients is still unknown. In addition, the involvement of at least 58 distinct genes sometimes makes the precise diagnosis difficult.

Targeted exon sequencing of selected genes using the Massively Parallel DNA Sequencing (MPS) technology will potentially enable us to systematically tackle previously intractable monogenic disorders and improve molecular diagnosis. We have recently reported that target exon sequencing using MPS is a powerful tool to identify rare gene mutations for deafness patients [4].

In this study, we have chosen 58 deafness-causative genes, and conducted genetic analysis using MPS-based genetic screening to find the rare genes responsible for the patients who received CI or EAS.

## Subjects and Methods

### Subjects

Eight deafness patients (4 early-onset, 4 late-onset) were randomly selected from among 150 CI or EAS patients (69 male and 81 female, aged 0 to 91), without common *GJB2, SLC26A4*, or mitochondrial 1555A>G or 3243A>G mutations determined by direct sequencing. Four patients with early-onset deafness received CI, and 4 late-onset patients had residual hearing at lower frequencies and received EAS. All subjects or next of kin, caretakers, or guardians on the behalf of the minors/children gave prior written informed consent for participation in the project, and the Ethical Committee of Shinshu University approved the study and the consent procedure.

Auditory behavioral development was assessed by IT-MAIS and LittlEARS, both of which are parent questionnaires regarding a young infant or toddler’s auditory behavior [5], [6]. IT-MAIS consists of 10 questions, each scored on a 5-point scale: 0 = never, 1 = rarely, 2 = occasionally, 3 = frequently, and 4 = always. LittlEARS has 35 questions, each scored as 1 = yes, and 0 = no.

### Amplicon Library Preparation

An Amplicon library of the target exons was prepared with an Ion AmpliSeq™ Custom Panel (Applied Biosystems, Life Technologies., Carlsbad, CA) designed with Ion AmpliSeq™ Designer (https://www.ampliseq.com/browse.action) for 58 genes reported to be causative of non-syndromic hearing loss listed in Table S1 (Hereditary Hearing loss Homepage; http://hereditaryhearingloss.org/) by using Ion AmpliSeq™ Library Kit 2.0 (Applied Biosystems, Life Technologies) and Ion Xpress™ Barcode Adapter 1–16 Kit (Applied Biosystems, Life Technologies) according to the manufacturers’ procedures.

In brief, DNA concentration was measured with Quant-iT™ dsDNA HS Assay (Invitrogen, Life Technologies) and Qubit® Fluorometer (Invitrogen, Life Technologies) and DNA quality was confirmed by agarose gel electrophoresis. 10 ng of each genomic DNA sample was amplified, using Ion AmpliSeq™ HiFi Master Mix (Applied Biosystems, Life Technologies) and AmpliSeq™ Custom primer pools, for 2 min at 99°C, followed by 15 two-step cycles of 99°C for 15 sec and 60°C for 4 min, ending with a holding period at 10°C in a PCR thermal cycler (Takara, Shiga, Japan). After the Multiplex PCR amplification, amplified DNA samples were digested with FuPa enzyme at 50°C for 10 min and 55°C for 10 min and the enzyme was successively inactivated for 60°C for 20 min incubation. After digestion, diluted barcode adapter mix including Ion Xpress™ Barcode Adapter and Ion P1 adaptor were ligated to the end of the digested amplicons with ligase in the kit for 30 min at 22°C and the ligase was successively inactivated at 60°C for 20 min incubation. Adaptor ligated amplicon libraries were purified with the Agencourt AMPure XP system (Beckman Coulter Genomics, Danvers, MA). The amplicon libraries were quantified by using Ion Library Quantitation Kit (Applied Biosystems, Life Technologies) and the StepOne plus realtime PCR system (Applied Biosystems, Life Technologies) according to the manufacturers’ procedures. After quantification, each amplicon library was diluted to 20pM and the same amount of the 6 libraries for 6 patients were pooled for one sequence reaction.

### Emulsion PCR and Sequencing

The emulsion PCR was carried out with the Ion OneTouch™ System and Ion OneTouch 200 Template Kit v2 (Life Technologies) according to the manufacturer’s procedure (Publication Part Number 4478371 Rev. B Revision Date 13 June 2012). After the emulsion PCR, template-positive Ion Sphere™ Particles were enriched with the Dynabeads® MyOne™ Streptavidin C1 Beads (Life Technologies) and washed with Ion OneTouch™ Wash Solution in the kit. This process were performed using an Ion OneTouch™ ES system (Life Technologies).

After the Ion Sphere Particle preparation, MPS was performed with an Ion Torrent Personal Genome Machine (PGM) system using the Ion PGM™ 200 Sequencing Kit and Ion 318™ Chip (Life Technologies) according to the established procedures (Publication Part Number 4474596 Rev. B Revision Date 14 July 2012).

### Base Call and Data Analysis

The sequence data were processed with standard Ion Torrent Suite™ Software and Torrent Server successively mapped to human genome sequence (build GRCh37/hg19) with Torrent Mapping Alignment Program optimized to Ion Torrent™ data. The average of 412.93 Mb sequences with about 3,200,000 reads was obtained by one Ion 318 chip. The 98.0% sequences were mapped to the human genome and 94.9% of them were on the target region. Average coverage of depth in the target region was 326.5 and 94.2% of them were over 20 coverage.

After the sequence mapping, the DNA variant regions were piled up with Torrent Variant Caller plug-in software. Selected variant candidates were filtered with the average base QV (minimum average base quality 25), variant frequency (40–60% for heterozygous mutations and 80–100% for homozygous mutations) and coverage of depth (minimum coverage of depth 10). After the filtrations, variant effects were analyzed with the wANNOVAR web site [7], [8] (http://wannovar.usc.edu) including the functional prediction software for missense variants listed below. PhyloP (http://hgdownload.cse.ucsc.edu/goldenPath/hg18/phyloP44way/), Sorting Intolerant from Tolerant (SIFT; http://sift.jcvi.org/), Polymorphism Phenotyping (PolyPhen2; http://genetics.bwh.harvard.edu/pph2/), LRT (http://www.genetics.wustl.edu/jflab/lrt_query.html), MutationTaster (http://www.mutationtaster.org/), and GERP++ (http://mendel.stanford.edu/SidowLab/downloads/gerp/index.html).

### Algorithm

**Figure 1:**
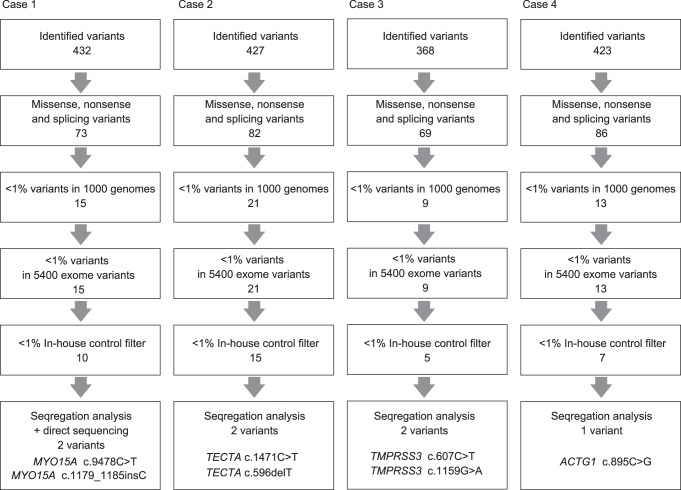
Flow of informatics analysis. Selected missense, nonsense, and splicing variants were filtered with 1) the 1000 genomes, 2) the 5400 exome variants, and 3) the in-house control. Responsible mutations were confirmed by segregation analysis.

Flow of informatics analysis is shown in Fig. 1. Missense, nonsense, and splicing variants were selected among the identified variants. Variants were further selected as less than 1% of, 1) the 1000 genome database (http://www.1000genomes.org/), 2) the 5400 exome variants (http://evs.gs.washington.edu/EVS/), and 3) the 72 in-house controls. Candidate mutations were confirmed by Sanger sequencing and the responsible mutations were identified by segregation analysis using samples from family members of the patients.

### Direct Sequence Analysis

Primers were designed with the Primer 3 plus web server (http://www.bioinformatics.nl/cgi-bin/primer3plus/primer3plus.cgi). Each genomic DNA sample (40 ng) was amplified using AmpliTaq Gold (Life Technologies) for 5 min at 94°C, followed by 30 three-step cycles of 94°C for 30 sec, 60°C for 30 sec, and 72°C for 30 sec, with a final extension at 72°C for 5 min, ending with a holding period at 4°C in a PCR thermal cycler (Takara, Shiga, Japan). The PCR products were treated with ExoSAP I (GE Healthcare Bio, Buckinghamshire, UK) and by incubation at 37°C for 30 min, and inactivation at 80°C for 15 min. After the products were purified, we performed standard cycle sequencing reaction with ABI Big Dye terminators in an ABI 3130×l sequencer (Life Technologies).

## Results

After informatics analysis, several candidate variants were identified and segregation analysis confirmed responsible mutations in *MYO15A* (Case #1) and *TECTA* (Case #2) in pre-lingual patients with conventional CI, and mutations in *TMPRSS3* (Case #3) and *ACTG1* (Case #4) were identified in patients with post-lingual deafness with EAS (Fig. 1). All detected mutations were predicted to be pathologic by several software programs (Table 1). In the remaining four cases, there were no conclusive causative mutations found in this study.

**Table 1:** Missense mutations found in this study.

Gene	Base Change	AA Change	ESP5400	1000g2012feb	dbSNP135	PhyloP	SIFT	PolyPhen2	LRT	MutationTaster	GERP++
*MYO15A*	c.9478C>T	p.L3160F	0.007618	0.01	rs140029076	N (0.885983)	D (0.97)	NA (0.754167)	NA (0.981216)	D (0.99518)	0.651
*TECTA*	c.1471C>T	p.R491C	–	–	–	C (0.998333)	D (0.97)	D (1)	D (1)	D (0.684828)	4.88
*TMPRSS3*	c.1159G>A	p.A387T	–	–	–	C (0.997807)	D (0.96)	B (0.074)	D (1)	N (0.364687)	4.62
*ACTG1*	c.895C>G	p.L299V	–	–	–	C (0.978424)	NA(0.750464)	B (0.006)	D (0.99998)	D (0.999635)	1.2

SIFT, Polyphen-2, PhyloP, LRT, Mutation Taster, and GERP++ are functional prediction scores in which increasing values indicate a probable mutation. ESP5400 and 100g2012feb are the allele frequency in each 5400 exome and 1000 genome project.

Abbreviations: C, conserved; N, not-conserved or neutral D, damaging or deleterious; B, benign; NA, not applicable.

### Case #1: Severe Hearing Loss caused by *MYO15A* Mutations (Fig. 2)

**Figure 2:**
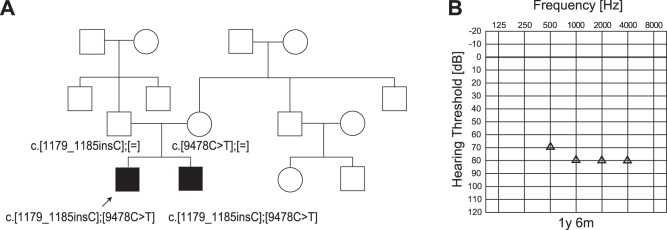
The CI patient with *MYO15A* mutations. A: The patient has compound heterozygous *MYO15A* mutations (c.[9478C>T]; [1179_1185insC]), and the parents were found to be carriers for these mutations. B: COR audiogram finding (1y 6 m).

As in Fig. 1, MPS identified 10 candidate variants in 9 genes. Among the 9 genes, *CDH23* and *MYO15A* are known to be inherited in a recessive manner. Sanger sequencing could not detect the *CDH23* variant. A *MYO15A* mutation (c.9478C>T (p.L3160F)) was confirmed by Sanger sequencing. Consecutive Sanger sequencing analysis identified another mutation, c.1179_1185insC, which was not found by MPS. The inconsistent results between the two methods were due to this mutation being located in the homo-polymer (poly C stretch) region, which is difficult to detect using this system [9] The patient (5y 5 m-old boy) had compound heterozygous *MYO15A* mutations (c.[9478C>T];[1179_1185insC]), and the parents were found to be carriers for these mutations (Fig. 2A). The frameshift mutation c.1179_1185insC, leading to a stop codon, was predicted to be causative, and the missense mutation, c.9478C>T, was predicted to be pathologic by several software programs (Table 1).

His hearing loss was found through newborn hearing screening using OAE. Auditory steady state response (ASSR) and conditioned orientation reflex (COR) evaluated at the ages of 1y 6 m, 2y 3 m, 2y 8 m, and 3y 6 m showed progressive hearing loss. He used hearing aids and some language development was seen, but due to progressive hearing loss, hearing aid amplification was insufficient, and he received a left CI (MEDEL PULSAR CI100/standard electrode) at the age of 4y 9 m. To obtain the final outcome, long-term follow up will be needed, but language was developed after 3 months of CI use (Scores of IT-MAIS: 16/40>25/40, LittlEar: 28>33).

### Case #2: Profound Hearing Loss caused by *TECTA* Mutations (Fig. 3)

**Figure 3:**
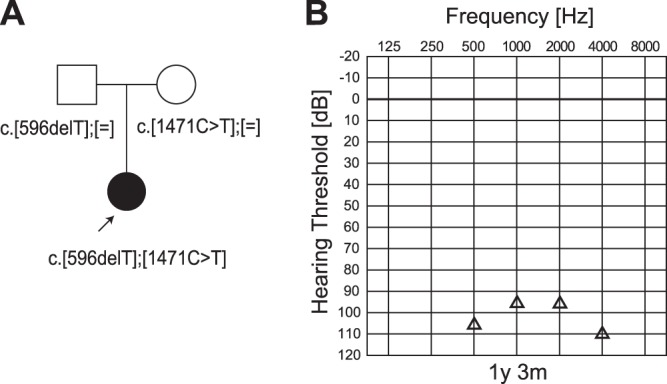
The CI patient with *TECTA* mutations. A: The patient has compound heterozygous *TECTA* mutations (c.[596delT];[1471C>T]), and the parents were found to be carriers for these mutations. B: COR audiogram finding (1y 9 m).

The patient (a 2-year-old boy) had compound heterozygous *TECTA* mutations (c.[596delT];[1471C>T]), and the parents were found to be carriers for these mutations (Fig. 3A). The frameshift mutation, c.596delT, leading to a stop codon, was predicted to be pathologic. The missense mutation, c.1471C>T (p.R491C), was predicted to be pathologic by several software programs (Table 1).

His hearing loss was found through newborn hearing screening using OAE. ASSR and COR evaluated at the age of 8 m, 1 y 3 m, and 1 y 9 m showed progressive hearing loss. He used hearing aids, but due to insufficient amplification, he received a left CI at the age of 2. Language was developed after 4 months of CI use (Scores of IT-MAIS: 9/40>23/40).

### Case#3: Late Onset Hearing Loss with Residual Hearing in Low Frequencies caused by *TMPRSS3* Mutations (Fig. 4)

**Figure 4:**
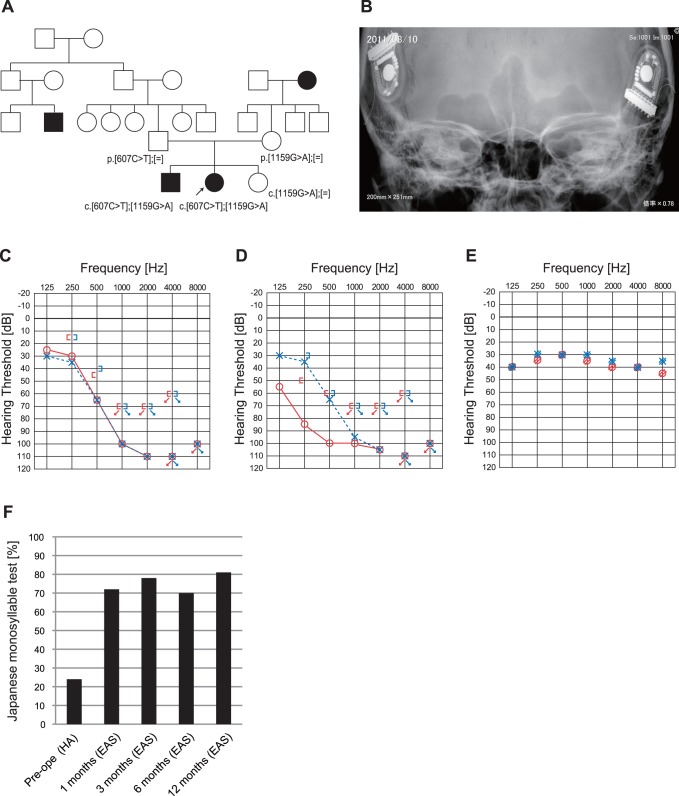
The EAS patient with *TMPRSS3* mutations. A: The patient has compound heterozygous *TMPRSS3* mutations, c.[607C>T];[1159G>A], and the parents were found to be carriers for these mutations. The patient’s brother also has the same mutations. B: X-ray imaging after bilateral EAS. C: Pre-operative audiogram. D: Post-operative audiogram (left: 24 months after first EAS, right: 4 months after second EAS). E: Hearing threshold with bilateral EAS. F: Japanese monosyllable test (65dB SPL in quiet) showing dramatic improvement with bilateral EAS.

The patient (a 40-year-old woman) had compound heterozygous *TMPRSS3* mutations c.[607C>T];[1159G>A] (p.[Q203X];[A387T]) (Fig. 4A). The nonsense mutation p.Q203X was predicted to be causative, and the missense mutation (p.A387T) was predicted to be pathologic by several software programs (Table 1). The parents were found to be carriers for these mutations. She had hearing loss detected by mass screening in primary school. It appeared to slowly progress, and by age 25 she suffered inconvenience in hearing and communication. EAS (MEDEL PULSAR FLEXeas) was applied at the ages of 38 and 39. Residual hearing for acoustic amplification could be preserved, and hearing level with bilateral EAS was around 30dB (Fig. 4C–E). Japanese monosyllable test (65dB SPL in quiet) showed dramatic improvement with bilateral EAS from 18% to 90% one year after receiving the second EAS (Fig. 4F).

### Case #4: Late Onset Hearing Loss with Residual Hearing in Low Frequencies caused by *ACTG1* Mutation (Fig. 5)

**Figure 5:**
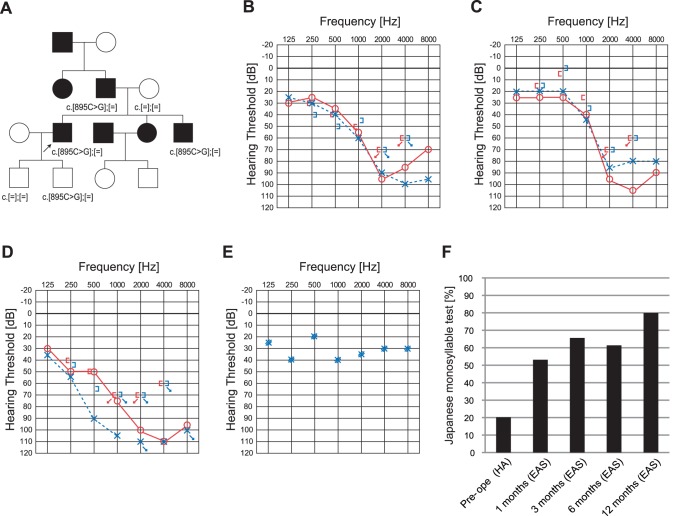
The EAS patient with *ACTG1* mutation. A: The patient has heterozygous *ACTG1* mutation, c.895C>G. Pedigree is compatible with autosomal dominant hearing loss. His father and brother carried the same mutation. B: Pre-operative audiogram. C: Audiogram of brother. D: Post-operative audiogram (6 months after EAS). E: Hearing threshold with EAS. F: Japanese monosyllable test (65dB SPL in quiet) showing dramatic improvement with EAS.

The patient (a 41-year-old man) had a heterozygous *ACTG1* mutation, c.895C>G (p.L299V) (Fig. 5A). His pedigree was compatible with autosomal dominant hearing loss. A missense mutation, p.L299V, was predicted to be pathologic by several software programs (Table 1). He noticed his hearing loss at around age 20. He received EAS due to progressive hearing loss. Residual hearing for acoustic amplification could be preserved, and hearing level with bilateral EAS was around 30dB (Fig. 5B, D, E). Japanese monosyllable test (65dB SPL in quiet) showed dramatic improvement from 20% to 80% one year after receiving EAS (Fig. 5F). His father and brother carried the same mutation. The audiogram of the brother is shown in Fig. 5C. His father also has hearing loss based on anamnestic evaluation. Neither of the patient’s sons (aged 10 and 12) have any hearing loss evaluated by pure tone audiogram, although the younger son has the same mutation.

## Discussion

The present MPS-based genetic analysis efficiently identified rare causative mutations in four genes, *MYO15A, TECTA, TMPRSS3,* and *ACTG1*. All except *TMPRSS3* were first reported in patients with CI/EAS.

*MYO15A* has been reported mainly in severe to profound hearing loss [10]. Therefore, it is not surprising the patient with the *MYO15A* mutation was found among the CI patients. However, probably due to being too large to be screened by conventional direct sequencing, the routine screening of this particular gene was hampered in spite of its importance in this particular population. *MYO15A* is known to be responsible for DFNB3 [11]. *Myosin 15a* localizes to the tips of inner ear sensory cell stereocilia and is essential for staircase formation of the hair bundle [12]. Since the etiology is located within the sensory hair cells, comparatively better outcomes can be predicted. This case in fact showed better performance after CI.

*TECTA* encodes α-tectorin, the major component of non-collagenous glycoprotein of the tectorial membrane. *TECTA* has been reported to be responsible for both autosomal dominant non-syndromic sensorineural hearing loss (ADNSHL) (DFNA8/12) and autosomal recessive non-syndromic sensorineural hearing loss (ARNSHL) (DFNB21). Dominant *TECTA* mutations can cause mid-frequency, high-frequency progressive HL, and *TECTA* is reported to be the commonest causative gene among ADNSHL [13]. Dominant inherited deafness caused by this gene has not been reported to reach the level of profound hearing loss. In contrast, recessive *TECTA* mutations cause more profound hearing loss [14]. The etiology is located within the cochlea, therefore comparatively better outcomes can be predicted. This is the first report of a patient with mutations in this gene showing good outcome as prospected from intra-membranous labyrinth etiology.

In this study, *TMPRSS3* was identified in a patient with post-lingual deafness with EAS (Case #3).

*TMPRSS3* is a member of the Type II Transmembrane Serine Protease family.

*TMPRSS3* may be involved in processing proneurotrophins and therefore in the development and survival of the cochlear neurons [15].

*TMPRSS3* has been reported to be responsible for DFNB8/10. Typically, the patients show ski-slope type audiograms and progressive HL [16], being compatible with the phenotype of the present patient. Outcome of CI for patients with *TMPRSS3* is controversial [2], [16], [17]. Two older papers reported good outcome of CI, while a recent report described poorer performance. Eppsteiner et al. [2] reported two cases of 58-year-old patients with a history of progressive hearing loss starting at the age of 5–6 years. Both of their outcomes were poorer compared with other patients, and the authors hypothesized that it was because the encoded protein is also expressed in the spiral ganglion. However, the present 40-year-old patient showed completely different performance after EAS, indicating that CI is not a contraindication and CI and/or EAS can be a recommended therapeutic option. Especially, the previously reported typical phenotype is high frequency involved hearing loss, which is a good indication for EAS. In the literature, there is also a severe phenotype with all frequencies affected [18]. Our 40-year-old patient did not have rapid progressive hearing loss (only 24 dB (125+250+500 Hz/3) during the 7-year follow-up period), supporting that this patient was a good candidate for EAS. Within this family, intra-familial variation was observed, i.e., an elder brother with the same mutations showed early onset (10 y.o.) profound hearing loss. Therefore, other factors may also potentially be involved in determining the phenotype (including severity and progression).

*ACTG1* was identified in a patient with post-lingual deafness with EAS (Case #4).

His brother (35 y.o.) also showed similar high frequency involved progressive hearing loss. Together with the previous literature, high frequency involved progressive nature is one of the characteristic features of the patients with *ACTG1* mutations. The present study proved that EAS is a good therapeutic option for the patients with this gene mutation. *ACTG1* is known to be responsible for DFNA20/26. *ACTG1*, encoding gamma-actin, is the predominant actin isoform in auditory hair cells, more specifically in the cuticular plate, adherens junctions and stereocilia [19]. The etiology is located within the cochlea, therefore comparatively better outcomes can be predicted. Our patient’s successful performance after EAS is compatible with the intra-membranous labyrinth etiology. The younger son who carried the same mutation will potentially have progressive hearing loss and his hearing is currently checked semiannually.

EAS is a new trend in therapy for the patients with residual hearing in the lower frequencies [20]. Various genes may be involved in the candidates [21], and we have found the mitochondrial 1555 A>G mutation and *CDH23* mutations in the patients receiving EAS [22], suggesting that the patients with those etiologies may have a potential to show good outcomes. Using the new MPS platform based on new generation sequencing enabled us to add two responsible genes, *TMPRSS3,* and *ACTG1*, in the patients with EAS. Identification of those genes may be good predictor when choosing the therapeutic options. Since the speed of progression may depend on the responsible gene, this information may be helpful for timing of EAS surgery and the selection of the electrode.

Overall, the current findings confirmed the importance of genetic information for predicting outcome of the CI/EAS patients, i.e., relatively good performance would be expected if the pathology exists within the cochlea. Such molecular diagnosis is important for the decision making process for selection of appropriate intervention, such as conventional cochlear implantation, EAS, hearing aid, or combination with other communication modes.

In spite of difficulty in discovery of the responsible gene for each individual patient, genetic testing using MPS may be a breakthrough. In the current series, MPS successfully discovered rare causative genes in CI patients and in EAS patients. These genes have not usually been screened and therefore mutations in them have not been diagnosed by the conventional approach. From that point of view, MPS has the potential power to identify such rare genes/mutations.

## Supporting Information

Table S1 58 genes reported to be causative of non-syndromic hearing loss.
(PDF)
