# Supplementary material for: Massively Parallel DNA Sequencing Successfully Identifies New Causative Mutations in Deafness Genes in Patients with Cochlear Implantation and EAS
Source: PLoS One. 2013 Oct 9;8(10):e75793. doi: 10.1371/journal.pone.0075793 (PMC3794008; doi:10.1371/journal.pone.0075793)
Supplement: Table S1 — 58 genes reported to be causative of non-syndromic hearing loss. (PDF) [file pone.0075793.s001.pdf]

Supplementary Table S1. 58 deafness-causative genes.

No	Locus Symbol	Gene Symbol	Gene ID	Transcript Variant
1	DFNA1	*DIAPH1*	NM_005219	NM_001079812
2	DFNA2	*KCNQ4*	NM_004700	NM_172163
3	DFNA2	*GJB3*	NM_024009	NM_001005752
4	DFNA3	*GJB6*	NM_006783	NM_001110219
				NM_001110220
				NM_001110221
5	DFNA4	*MYH14*	NM_024729	NM_001145809.
				NM_001077186
6	DFNA5	*DFNA5*	NM_004403	NM_001127454
				NM_001127453
7	DFNA6/14/38	*WFS1*	NM_006005	NM_001145853
8	DFNA8/12/DFNB21	*TECTA*	NM_005422	-
9	DFNA9/31	*COCH*	NM_004086	NM_001135058
10	DFNA10	*EYA4*	NM_004100	NM_172103
				NM_172105
11	DFNA11/DFNB2/USH1B	*MYO7A*	NM_000260	NM_001127179
				NM_001127180
12	DFNA13/DFNB53/STL3	*COL11A2*	NM_080680	NM_080679
				NM_080681
				NM_001163771
13	DFNA15	*POU4F3*	NM_002700	-
14	DFNA17	*MYH9*	NM_002473	-
15	DFNA20/DFNA26	*ACTG1*	NM_001614	NM_001199954
16	DFNA22/DFNB37	*MYO6*	NM_004999	-
17	DFNA25	*SLC17A8*	NM_139319	NM_001145288
18	DFNA28	*GRHL2*	NM_024915	-
19	DFNA36/DFNB7/11	*TMC1*	NM_138691	-
20	DFNA40	*CRYM*	NM_001888	NM_001014444
21	DFNA44	*CCDC50*	NM_178335	NM_174908
22	DFNA48	*MYO1A*	NM_005379	-

23	DFNA51	*TJP2*	NM_001170414	NM_004817
				NM_201629
				NM_001170630
				NM_001170415
				NM_001170416
24	DFNA64	*DIABLO*	NM_019887	NM_138929
25		*CEACAM16*	NM_001039213	-
26	DFNB1/DFNA3	*GJB2*	NM_004004	-
27	DFNB3	*MYO15A*	NM_016239	-
28	DFNB4/Pendred	*SLC26A4*	NM_000441	-
29	DFNB6	*TMIE*	NM_147196	-
30	DFNB8/10	*TMPRSS3*	NM_024022	NM_032405
31	DFNB9	*OTOF*	NM_194248	NM_194322
				NM_194323
				NM_004802
32	DFNB12/USH1D	*CDH23*	NM_022124	NM_001171930
				NM_001171931
				NM_001171932
				NM_001171933
				NM_001171934
				NM_001171936
				NM_052836
33	DFNB15/72/95	*GIPC3*	NM_133261	-
34	DFNB16	*STRC*	NM_153700	-
35	DFNB18/USH1C	*USH1C*	NM_153676	NM_005709
36	DFNB22	*OTOA*	NM_144672	NM_001161683
				NM_170664

37	DFNB23/USH1F	*PCDH15*	NM_033056	NM_001142763
				NM_001142764
				NM_001142765
				NM_001142766
				NM_001142767
				NM_001142769
				NM_001142770
				NM_001142771
				NM_001142772
				NM_001142773
38	DFNB24	*RDX*	NM_002906	-
39	DFNB25	*GRXCR1*	NM_001080476	-
40	DFNB28	*TRIOBP*	NM_007032	NM_138632
				NM_001039141
41	DFNB29	*CLDN14*	NM_144492	NM_001146077
				NM_001146078
				NM_001146079
				NM_012130
42	DFNB30	*MYO3A*	NM_017433	-
43	DFNB31/USH2D	*WHRN*	NM_015404	
44	DFNB35	*ESRRB*	NM_004452	-
45	DFNB36	*ESPN*	NM_031475	-
46	DFNB39	*HGF*	NM_000601	NM_001010931
				NM_001010932
				NM_001010933
				NM_001010934
47	DFNB42	*ILDR1*	NM_001199799	NM_001199800
				NM_175924
48	DFNB49	*MARVELD2*	NM_001038603	NM_001244734
49	DFNB59	*PJVK*	NM_001042702	
50	DFNB61	*SLC26A5*	NM_206883	NM_001167962
				NM_206884

				NM_206885
				NM_198999
51	DFNB63	*LRTOMT*	NM_001145307	NM_001145308
				NM_001205138
				NM_145309
52	DFNB66/67	*LHFPL5*	NM_182548	-
53	DFNB74	*MSRB3*	NM_198080	NM_001031679
				NM_001193460
				NM_001193461
54	DFNB77	*LOXHD1*	NM_144612	NM_001173129
				NM_001145472
				NM_001145473
55	DFNB79	*TPRN*	NM_001128228	-
56	DFNB82	*GPSM2*	NM_013296	-
57	DFNB84	*PTPRQ*	NM_001145026	-
58	DFNB91	*SERPINB6*	NM_004568	NM_001195291
